# Supplementary material for: Image quality improvement of liver ultrasound using unsupervised deep learning
Source: PLoS One. 2026 Apr 28;21(4):e0348137. doi: 10.1371/journal.pone.0348137 (PMC13123951; doi:10.1371/journal.pone.0348137)
Supplement: Attachment — Submitted filename: Response to Reviewers_PLOS.docx [file pone.0348137.s001.docx]

August 28th, 2024

Editorial office***, PLOS ONE***

We would like to thank you and the reviewers for the valuable and constructive comments offered on our previous submission entitled " Image quality improvement of liver ultrasound using unsupervised deep learning" to the ***PLOS ONE***. We prepared a point-by-point response to the reviewers’ comments. We tried to accept all the editor and reviewers' suggestions and answer all queries. Our specific responses to indications and suggestions are as follows:

***To the comments of Reviewer 1***

1. Improve the quality of Fig 2. The text is not in readable form.

Thank you for your comments. As reviewer 1’s comment, we have improved the image quality of Figure 2 as you suggested, and I have adjusted the font size. Thank you for your thoughtful comment.

2. Verify that the format of the abstract is according to the journal format.

Thank you for your comments. We revised the format of the abstract adheres to the journal’s guide lines. Thank you for your attention to detail and grateful for the opportunity to ensure that our submission meets the required standards

3. References format is not according to the journal format.

Thank you for your descent comments. Following the reviewer’s advice, we have revised the references.

4.  Improved the Flow chart of liver ultrasound dataset preparation.

Thank you for your comments. We revised our flow chart of the liver ultrasound dataset preparation to enhance its visibility and clarity as follow.:

5. Some experiments will be done which show the results before and after Image quality improvement of liver ultrasound.

Thank you for your comments. From our understanding, we have to do some comparison experiments and add the results of it with the proposed method results. Therefore, we have added Figure 8, which includes our additional results along with those from other comparison experiments. If we have misunderstood your focus, please let us know, and we will make further revisions accordingly.

6. How does the proposed unsupervised deep learning approach compare with existing methods for liver ultrasound image enhancement

Thank you for your comments. Conventional liver ultrasound image enhancement methods rely on various image filters. To verify that our proposed method is superior, we implemented the shark and bilateral filters, as used in references (43, 44), and included the results in Figure 8. Our method is based on the image-to-image (I2I) translation approach, which has been widely researched. We conducted a comparative experiment using a contrastive-based learning method for efficient I2I, training the open-source code provided by the authors on the same dataset as our method. The results are discussed in the section 'Comparison with Other Methods', ’Algorithm comparison’ section. For a quantitative evaluation, we calculated the CR and CNR values to assess contrast enhancement and the Frechet Inception Distance (FID) score to measure the distance between the generated images and real target images in the feature domain. Our results show that the proposed method performs similarly to or better than other methods both qualitatively and quantitatively.

7. Need to enhanced the Introduction section.

Thank you for your comments. We have refined the introduction section through the following sentences to enhance its comprehensibility.

8. Can you provide a more detailed explanation of the network architecture used in the study?

Thank you for your comments. In ‘Swithcable cycle generative adversarial network’ section, we revised and added more details about the entire framework of the Switchable CycleGAN and the generator architecture, including Adaptve Instance Normalization (AdaIN) Code Generator (ACG).

***To the comments of Reviewer 2***

The manuscript does not adhere to our journal's formatting guidelines. It has been found that the figures and tables placement within the manuscript is incorrect, and the captions are improperly formatted. References are not according properly. Additionally, the quality and resolution of the figures do not meet our publication standards. Need to improve the abstract, introduction section, and methodology. Need to add some more discussion about the background and existing work. Add the dataset description in tabular form. Some Mathematical equations have been used but do not mention the equation number. Add some more details about how to utilize the Switchable cycle generative adversarial network. As the proposed method was evaluated by visual inspection, is it better some other way will be used for the analysis and evaluated the images?

The abstract, introduction and method sections was revised to enhanced clarity and comprehensibility. We have expanded the discussion on existing work and background to provide more thorough context. A table summarizing the dataset preparation was added to the manuscript.

Evaluation methods for ultrasound image quality can vary widely. However, the assessment of image quality itself may not always align with clinical significance. Therefore, our study posits that evaluations performed through visual assessment by experienced radiologists, especially abdominal radiologists directly involved in clinical practice hold greater relevance.

1. The manuscript does not adhere to our journal's formatting guidelines. It has been found that the figures and tables placement within the manuscript is incorrect, and the captions are improperly formatted. References are not according properly. Additionally, the quality and resolution of the figures do not meet our publication standards.

Thank you for your descent comments. We revised the manuscript to ensure compliance with the guidelines. The placement of graphs and tables has been corrected, and the caption formatting adjusted accordingly. Also, we properly formatted the references to align with the requirements. The quality and resolution of the figures was improved to meet the publication standards.

2. Need to improve the abstract, introduction section, and methodology.

Thank you for your descent comments. The abstract, introduction and method sections was revised to enhanced clarity and comprehensibility.

3. Need to add some more discussion about the background and existing work.

Thank you for the constructive comments. We added contents about existing work about medical image quality enhancement and image translation method in deep learning technique on ‘Introduction’ section.

4. Add the dataset description in tabular form.

Thank you for your comments. We added a table for dataset in the Material and Methods section to aid in its comprehension. Should there be any point for improvement, we will make the necessary revision. Thank you again your comment.

5. Some Mathematical equations have been used but do not mention the equation number.

Thank you for comments. We added the equation number.

6. Add some more details about how to utilize the Switchable cycle generative adversarial network.

Thank you for valuable comment. In ‘Switchable cycle generative adversarial network’ section, we added and revised more information about the Switchable CycleGAN framework.

7. As the proposed method was evaluated by visual inspection, is it better some other way will be used for the analysis and evaluated the images?

Thank you for the constructive comments. One of our key metrics for image quality assessment is contrast. Therefore, we included the Contrast Ratio (CR) and Contrast Noise Ratio (CNR) values for a quantitative comparison between the proposed method and other methods. We selected foreground and background regions in the images and calculated these values, averaging the results over 100 test sets, as shown in Table 6. Since our proposed method is trained in an unsupervised manner and does not use an exact paired dataset, we were unable to perform a direct quantitative evaluation. However, in image style transfer research, the Frechet Inception Distance (FID) score is commonly used to evaluate how closely the network's output resembles real target images. The FID score measures the distribution distance between real and generated images. For this study, we utilized 1,500 real high-quality images and 1,500 generated high-quality images to calculate the FID score, with the results presented in Table 5. Note that the FID score was utilized only for I2I translation comparison methods.

Thank you again for the valuable comments of the reviewers. We did our best for the revision to enhance our manuscript's quality. We hope that the revised version of the manuscript meets with your and reviewer’s approval and will be considered for publication in the ***PLOS ONE***

Sincerely yours,

Eun Sun Lee, MD, PhD
