# Supplementary material for: Image quality improvement of liver ultrasound using unsupervised deep learning
Source: PLoS One. 2026 Apr 28;21(4):e0348137. doi: 10.1371/journal.pone.0348137 (PMC13123951; doi:10.1371/journal.pone.0348137)
Supplement: Attachment — Submitted filename: PONE-D-23-32222_251013.docx [file pone.0348137.s002.docx]

**Manuscript #PONE-D-23-32222R1**

**Manuscript title:** Image quality improvement of liver ultrasound using unsupervised deep learning

**Summary:**
This manuscript by Huh et al. presents a deep learning approach using a Switchable CycleGAN model to enhance low-quality liver ultrasound images obtained from an older scanner, using high-quality images from a newer device as the target domain. The authors report that the algorithm improves brightness, contrast, and overall image quality, as evaluated by two radiologists, and modestly increases the sensitivity for detecting liver cirrhosis. The study addresses an important technical challenge of image quality heterogeneity among ultrasound devices and introduces an unsupervised learning framework that eliminates the need for paired datasets. The manuscript is generally well organized and technically sound. However, several aspects regarding the intended clinical application and translational significance of this work remain unclear and should be clarified to avoid potential overinterpretation.

**Comments**

1. The manuscript does not clearly specify whether the proposed algorithm is intended for retrospective post-hoc enhancement of previously acquired ultrasound images or for real-time enhancement during ongoing image acquisition. Based on the study design, dataset composition, and evaluation procedure, the present work clearly represents a retrospective post-processing approach. All data used for training and testing were previously collected static ultrasound images obtained between 2016 and 2018 from an older system, and the performance evaluation was conducted by comparing original and AI-processed images offline. Therefore, the algorithm has not been validated or demonstrated in a real-time or prospective scanning setting.

However, several sentences in the abstract and discussion—particularly the claim that the algorithm "facilitates early diagnosis and intervention for liver cirrhosis” could be interpreted as implying real-time diagnostic benefit. This creates a conceptual inconsistency between the actual experimental setup and the implied clinical utility. The authors should explicitly clarify that the current study was conducted retrospectively on stored static images and that real-time clinical application remains a future goal requiring further technical development, such as model optimization for inference speed, hardware integration, and prospective validation. Clear distinction between retrospective enhancement and potential real-time deployment is essential to properly contextualize the clinical relevance of this work.

2. The manuscript highlights a >10% increase in sensitivity for liver cirrhosis but fails to address the concurrent 15-16% decrease in specificity. With no significant improvement in overall diagnostic accuracy (ROC curves), this trade-off could lead to more false positives, undermining the claim of clinical benefit. The authors should expand the Discussion to critically address this sensitivity-specificity trade-off, acknowledging that the reported sensitivity gain does not necessarily translate to improved diagnostic accuracy or patient outcomes.

3. The manuscript lacks a discussion of the algorithm's limitations by only presenting successful outcomes. To provide a more balanced and clinically relevant assessment, the authors should include a failure analysis, discussing and showing examples where the image enhancement was suboptimal or failed, particularly in cases with severe artifacts or challenging patient anatomy.

4. The evaluation of image quality relies heavily on subjective radiologist scores. Objective metrics (CR, CNR) were used only for algorithmic comparison, not for validating the clinical test images. Crucially, the study lacks objective proof that the GAN preserves diagnostic vital parenchymal textures, which is a key concern for AI-generated medical images.

5. The model's applicability is highly limited as it was trained on only one specific pair of ultrasound machines (Aloka to Canon). It functions more as a device-to-device translator than a universally applicable enhancement tool. Its performance on equipment from other vendors is unproven and likely poor.

6. While the authors state that test datasets were "randomly shuffled," it is unclear whether the radiologists were blinded to image type (original vs. post-processed). Visual characteristics introduced by the GAN might allow experienced readers to identify processed images, potentially biasing subjective quality ratings. The authors should clarify the blinding procedure or, if blinding was not ensured, acknowledge this as a limitation.
