# Supplementary material for: Image quality improvement of liver ultrasound using unsupervised deep learning
Source: PLoS One. 2026 Apr 28;21(4):e0348137. doi: 10.1371/journal.pone.0348137 (PMC13123951; doi:10.1371/journal.pone.0348137)
Supplement: Attachment — Submitted filename: Response_to_Reviewers_PLOS_auresp_2.docx [file pone.0348137.s003.docx]

August th, 2024

Editorial office***, PLOS ONE***

We would like to thank you and the reviewers for the valuable and constructive comments offered on our previous submission entitled " Image quality improvement of liver ultrasound using unsupervised deep learning" to the ***PLOS ONE***. We prepared a point-by-point response to the reviewers’ comments. We tried to accept all the editor and reviewers' suggestions and answer all queries. Our specific responses to indications and suggestions are as follows:

***To the comments of Reviewer 3***

1. Heading after heading is not a good approach add content after each heading that covers what are you discussing in section: Like heading. Background (add content) then start next subheadings

Thank you for this helpful comment.

In the revised manuscript, we have clarified the motivation of our study and explicitly described why this work is important and which specific problems it aims to address.

First, in the “Background of Image-to-Image translation” subsection, we added a closing sentence that links existing I2I methods to the need for real-time medical imaging applications, such as ultrasound systems. This sentence explains that current diffusion-based approaches, although powerful, are computationally expensive, thereby motivating more efficient I2I methods for real-time use.

Second, between the I2I background and the “Background of medical image quality enhancement” subsection, we inserted a bridging sentence to clearly indicate that our focus narrows from general I2I methods to medical image quality enhancement, with a particular emphasis on ultrasound imaging.

Third, in the “Background of medical image quality enhancement” subsection, we added a short paragraph at the end that:

2. Quality of the images, particularly Fig 2, is inadequate. Both reviewers have noted this issue, and improvements are necessary to ensure that all figures are clearly readable.

We sincerely thank the reviewers for highlighting this issue. We fully agree that the resolution and readability of the figure were insufficient. Accordingly, all figures have been regenerated at 330 dpi (up from the original 96 dpi) to ensure clear and high-quality presentation.

3. The motivation behind this study is not clearly articulated. It is essential to express why this research is significant and what specific problems it aims to address.

Thank you for your valuable comment. In the revised manuscript, we have clarified the motivation of our study.

Specifically, in the Background section, we now explain that existing diffusion-based image-to-image translation methods are often too slow for real-time medical imaging applications such as ultrasound systems. We also describe the clinical challenges of liver ultrasound imaging (severe speckle noise and low contrast) and the lack of methods specifically targeting liver ultrasound with real-time applicability. Finally, we clearly state that the aim of our study is to develop an image-to-image translation–based deep learning method that enhances liver ultrasound image quality while preserving anatomical structures and enabling real-time processing, and to verify its clinical relevance through evaluation by two board-certified clinicians.

4. To strengthen the introduction, please include a bullet-point list of the study's contributions before concluding this section. This will help readers understand the unique aspects of your work.

Thank you for this constructive suggestion. We agree that clearly summarizing the contributions can help readers better grasp the unique aspects of our work.

In the current revision, we carefully reorganized the Introduction so that the clinical motivation, methodological novelty, and main objectives are already stated explicitly in the final paragraphs of the section. After testing several versions, we found that adding an additional bullet-point list of contributions led to substantial repetition of content that had just been described in the Background and motivation paragraphs. For this reason, we decided not to insert a separate bullet list in the main text, in order to keep the Introduction concise and avoid redundancy.

Nevertheless, we have ensured that the key contributions of the study are stated clearly and can be easily identified by the reader in the concluding part of the Introduction and in the Discussion section, where they are summarized again in the context of existing work.

The bullet-point list for example:

‘We propose a novel deep learning–based image-to-image translation method (CycleGAN variant) for enhancing the quality of liver ultrasound (US) images acquired with old or low-performance equipment.’

‘Our method directly addresses the challenges of severe speckle noise and low contrast that hinder reliable detection of liver lesions and is specifically designed for real-time clinical settings.’

‘We show that unsupervised learning with high-end equipment data can enable quality enhancement for low-end devices, without the need for paired datasets.’

‘To our knowledge, this is the first study to demonstrate deep learning–based image enhancement of poor-quality liver US by learning from high-end equipment, validated with input from two board-certified clinicians in real clinical cases.’

5. Clearly articulate the research gap that this study intends to fill. This will enhance the clarity and purpose of the study.

Thank you for this important comment. In the revised manuscript, we have explicitly articulated the research gap that our study aims to address.

Based on the existing literature summarized in the Introduction, we now clarify that (i) most deep learning–based enhancement methods have focused on CT, MRI, or specific ultrasound tasks such as segmentation and lesion classification, rather than directly improving the quality of poor-quality liver ultrasound images acquired with old or inadequately maintained equipment, and (ii) unsupervised image-to-image translation approaches such as CycleGAN have rarely been used to transfer image quality from high-end ultrasound systems to low-end devices in a clinically realistic setting.

We then state explicitly that our study fills this gap by proposing an unsupervised CycleGAN-based framework that learns from high-end liver ultrasound images to enhance low-quality liver ultrasound acquired with old equipment, and by evaluating its clinical relevance with assessments by board-certified clinicians. We believe these additions enhance the clarity and purpose of the study by making the targeted research gap more explicit.

6. After recommendations of both reviewers, I am still on to improve introduction section. The introduction is not strong and can be improved.

Thank you for this important remark regarding the overall strength of the Introduction.

Following the recommendations of both reviewers, we have substantially revised and strengthened the Introduction section. Specifically, we (i) clarified the clinical background and burden of chronic liver disease and the importance of liver ultrasound imaging, (ii) described in more detail the limitations of poor-quality liver ultrasound images obtained from old or inadequately maintained equipment, (iii) summarized recent advances in image-to-image translation and medical image quality enhancement, and (iv) explicitly stated the research gap and the main objective of our study in the concluding paragraphs of the Introduction. Given these revisions, we believe that the current version of the Introduction more clearly presents the context, research gap, and purpose of our work, and is now sufficiently strengthened while avoiding unnecessary repetition of content.

7. A dedicated section on related work should be added to explore various methodologies for improving detection quality from liver ultrasound images, explore from traditional methods machine learning methods to advanced deep learning including transfer learning.

Thank you for this valuable suggestion. In the revised manuscript, we have added a dedicated Related Work section that systematically reviews classical approaches, traditional filtering-based ultrasound enhancement techniques, machine-learning methods, and recent deep-learning developments, including transfer-learning–based models.

8. The related work section lacks references to recent studies (2023 and 2024). It is crucial to include and discuss these works to demonstrate the current landscape of research in this area.

Following the reviewer’s recommendation, we have substantially expanded the Related Work section to include and discuss recent studies published in 2023 and 2024 on ultrasound image quality enhancement and liver disease detection.

9. Please include a comparison of your proposed results with recent existing studies. This will provide context for the significance of your findings.

Thank you for this helpful suggestion. In the revised manuscript, we have incorporated two additional recent studies (published in 2023 and 2024) based on GAN and GAN–transformer architectures. The qualitative comparisons are presented in Figure 8, and the corresponding quantitative results are provided in Tables 5 through 7.

10. There should be a separate section for conclusions and future directions. In this section, clearly state the limitations of your work to provide a balanced view of your research.

Thank you for this helpful comment. In the revised manuscript, we have created a separate section entitled “Conclusions and future directions.” In this section, we clearly restate the main findings of our study, namely that the proposed unsupervised CycleGAN-based algorithm can enhance low-quality liver ultrasound images to higher quality while supporting earlier diagnosis and intervention for liver cirrhosis.

***To the comments of Reviewer 4***

1. The manuscript does not clearly specify whether the proposed algorithm is intended for retrospective post-hoc enhancement of previously acquired ultrasound images or for real-time enhancement during ongoing image acquisition. Based on the study design, dataset composition, and evaluation procedure, the present work clearly represents a retrospective post-processing approach. All data used for training and testing were previously collected static ultrasound images obtained between 2016 and 2018 from an older system, and the performance evaluation was conducted by comparing original and AI-processed images offline. Therefore, the algorithm has not been validated or demonstrated in a real-time or prospective scanning setting.
However, several sentences in the abstract and discussion—particularly the claim that the algorithm "facilitates early diagnosis and intervention for liver cirrhosis” could be interpreted as implying real-time diagnostic benefit. This creates a conceptual inconsistency between the actual experimental setup and the implied clinical utility. The authors should explicitly clarify that the current study was conducted retrospectively on stored static images and that real-time clinical application remains a future goal requiring further technical development, such as model optimization for inference speed, hardware integration, and prospective validation. Clear distinction between retrospective enhancement and potential real-time deployment is essential to properly contextualize the clinical relevance of this work.

We thank the reviewer for highlighting this important point. We agree that the present study is entirely retrospective and that real-time clinical deployment was not evaluated. In response, we have revised the Abstract, Introduction, and Discussion sections to explicitly state this limitation.

2.The manuscript highlights a >10% increase in sensitivity for liver cirrhosis but fails to address the concurrent 15-16% decrease in specificity. With no significant improvement in overall diagnostic accuracy (ROC curves), this trade-off could lead to more false positives, undermining the claim of clinical benefit. The authors should expand the Discussion to critically address this sensitivity-specificity trade-off, acknowledging that the reported sensitivity gain does not necessarily translate to improved diagnostic accuracy or patient outcomes.

Thank you for this insightful comment. We agree that the reported >10% increase in sensitivity for liver cirrhosis should be interpreted in the context of the concurrent 15–16% decrease in specificity and the lack of a significant change in overall diagnostic accuracy (ROC curves).

In the revised Discussion, we have added a dedicated paragraph explicitly addressing this sensitivity–specificity trade-off. We now state that the gain in sensitivity may come at the cost of more false-positive findings and that, therefore, the improvement does not automatically imply better overall diagnostic performance or improved patient outcomes. We also clarify that the primary aim of this study was to demonstrate the technical and clinical feasibility of unsupervised deep learning–based image quality enhancement for liver ultrasound, and that identifying an optimal operating point that better balances sensitivity and specificity will require further investigation in future studies.

3.The manuscript lacks a discussion of the algorithm's limitations by only presenting successful outcomes. To provide a more balanced and clinically relevant assessment, the authors should include a failure analysis, discussing and showing examples where the image enhancement was suboptimal or failed, particularly in cases with severe artifacts or challenging patient anatomy.

Thank you for this important comment. We agree that presenting only successful outcomes may give an overly optimistic impression and that a failure analysis is necessary for a balanced and clinically relevant assessment.

In the revised Discussion, we have added a dedicated paragraph describing cases in which the proposed algorithm did not perform optimally. Specifically, we now discuss patients with severe near-field reverberation artifacts or marked rib shadowing, in whom the enhancement sometimes failed to sufficiently reduce artifacts or even slightly accentuated noise along the liver surface. We also mention obese patients and those with markedly heterogeneous liver parenchyma, where improvements in brightness and contrast did not always translate into clearer visualization of surface nodularity or vascular structures. Furthermore, we indicate that these failure patterns suggest reduced robustness of the model under extreme artifacts and challenging anatomy, and that additional artifact-aware training and further refinement will be required before wider clinical application.

4. The evaluation of image quality relies heavily on subjective radiologist scores. Objective metrics (CR, CNR) were used only for algorithmic comparison, not for validating the clinical test images. Crucially, the study lacks objective proof that the GAN preserves diagnostic vital parenchymal textures, which is a key concern for AI-generated medical images.

Thank you for this valuable comment. To address the concern regarding the lack of objective evidence that the GAN preserves diagnostic parenchymal textures, we added a comprehensive structure-preservation evaluation (Table 7). This table reports patch-wise SSIM, first-order statistics (mean intensity, standard deviation, skewness, entropy), and second-order GLCM texture features for the proposed method and all comparison models.

These metrics are computed between the original input images and their enhanced versions, providing objective, quantitative measurements of texture stability in the clinical test set. The added table now demonstrates how closely the proposed method maintains key parenchymal characteristics, complementing the radiologists’ subjective assessment.

5.The model's applicability is highly limited as it was trained on only one specific pair of ultrasound machines (Aloka to Canon). It functions more as a device-to-device translator than a universally applicable enhancement tool. Its performance on equipment from other vendors is unproven and likely poor.

Thank you for this important comment. We agree that the current model was trained on a single pair of ultrasound machines (Aloka → Canon), and therefore its generalizability to other vendors has not been evaluated. We have now explicitly stated this limitation in the Discussion. Although the present work demonstrates a device-to-device translation setting, the underlying CycleGAN-based framework can be extended to additional ultrasound systems when corresponding training data are available.

6.While the authors state that test datasets were "randomly shuffled," it is unclear whether the radiologists were blinded to image type (original vs. post-processed). Visual characteristics introduced by the GAN might allow experienced readers to identify processed images, potentially biasing subjective quality ratings. The authors should clarify the blinding procedure or, if blinding was not ensured, acknowledge this as a limitation.

We appreciate this valuable comment. We completely agree with your point and have explicitly acknowledged it as a limitation in the revised manuscript.

Thank you again for the valuable comments of the reviewers. We did our best for the revision to enhance our manuscript's quality. We hope that the revised version of the manuscript meets with your and reviewer’s approval and will be considered for publication in the ***PLOS ONE***

Sincerely yours,

Joo Hyeok Choi, MD; Jaeyoung Huh PhD; Eun Sun Lee, MD, PhD
