# Supplementary material for: Image quality improvement of liver ultrasound using unsupervised deep learning
Source: PLoS One. 2026 Apr 28;21(4):e0348137. doi: 10.1371/journal.pone.0348137 (PMC13123951; doi:10.1371/journal.pone.0348137)
Supplement: Attachment — Submitted filename: PONE-D-23-32222_251231.docx [file pone.0348137.s004.docx]

Manuscript #PONE-D-23-32222R2

Manuscript title: Image quality improvement of liver ultrasound using unsupervised deep learning

Summary: The authors have adequately addressed all major concerns raised in the previous review. The retrospective nature of the study is now clearly stated, the sensitivity-specificity trade-off is critically discussed, failure analysis has been added, objective texture preservation metrics are provided, and limitations regarding single-device training and blinding are acknowledged. The manuscript is now suitable for publication pending minor corrections.

**Minor Comments**

1. In Table 6, the note states "Higher CR, CNR value represents better performance," which was copied from Table 5. This is incorrect for FID scores, where lower values indicate better performance. Please correct the note to accurately reflect the metric interpretation.

2. In Table 7, the column header "NEGCUT" appears to be truncated as "NEGCU." Please verify and correct the column header.

3. In the Conclusion section, the phrase "thereby helping clearer visualization" is grammatically awkward. Consider revising to "thereby enabling clearer visualization" or "thereby helping achieve clearer visualization."

4. References 46–49 lack complete bibliographic information (e.g., page numbers). Please ensure these references conform to the journal's citation format requirements.

5. In the Abstract, the phrase "supporting more accurate assessment" may still overstate the findings, given that overall diagnostic accuracy (ROC curves) did not significantly improve. The authors may consider a more conservative phrasing such as "potentially supporting clinical assessment" or "improving image quality that may support clinical interpretation."
