# Supplementary material for: Image quality improvement of liver ultrasound using unsupervised deep learning
Source: PLoS One. 2026 Apr 28;21(4):e0348137. doi: 10.1371/journal.pone.0348137 (PMC13123951; doi:10.1371/journal.pone.0348137)
Supplement: Attachment — Submitted filename: Response to Reviewers_PLOS_v3.docx [file pone.0348137.s005.docx]

Editorial office***, PLOS ONE***

We would like to thank you and the reviewers for the valuable and constructive comments offered on our previous submission entitled " Image quality improvement of liver ultrasound using unsupervised deep learning" to the ***PLOS ONE***. We prepared a point-by-point response to the reviewers’ comments. We tried to accept all the editor and reviewers' suggestions and answer all queries. Our specific responses to indications and suggestions are as follows:

***To the comments of Reviewer 4***

1. In Table 6, the note states "Higher CR, CNR value represents better performance," which was copied from Table 5. This is incorrect for FID scores, where lower values indicate better performance. Please correct the note to accurately reflect the metric interpretation.

Thank you for the comment. The note has been revised to accurately reflect the interpretation of the metric.

2. In Table 7, the column header "NEGCUT" appears to be truncated as "NEGCU." Please verify and correct the column header.

Thank you for the comment. We have revised it.

3. In the Conclusion section, the phrase "thereby helping clearer visualization" is grammatically awkward. Consider revising to "thereby enabling clearer visualization" or "thereby helping achieve clearer visualization."

Thank you for the careful comment. We have changed it into “thereby enabling clearer visualization”.

4.  References 46–49 lack complete bibliographic information (e.g., page numbers). Please ensure these references conform to the journal's citation format requirements.

Thank you for the comment. We have revised it in more detail.

5. In the Abstract, the phrase "supporting more accurate assessment" may still overstate the findings, given that overall diagnostic accuracy (ROC curves) did not significantly improve. The authors may consider a more conservative phrasing such as "potentially supporting clinical assessment" or "improving image quality that may support clinical interpretation."

Thank you for the careful comment. We have changed it into “potentially supporting clinical assessment”.

***To the comments of Reviewer 5***

1. Regarding the data source, it is unclear if the analysis used raw ultrasound data or images retrieved from the PACS system. If the study used PACS images, the compression levels might differ between the older and newer machines. This could affect the consistency of the training data. Please clarify the technical standards for image storage in the methods section.

Thank you for highlighting the need to clarify the image source and storage standards. In this retrospective study, all images were obtained from archived clinical ultrasound examinations exported in a consistent manner (not raw RF data). We have revised the Dataset section to clearly describe the data source (PACS/exported images), file format and bit depth, and archival compression policy. We also address the potential impact of vendor- or time-dependent compression differences and specify the measures taken to minimize bias, including a consistent export pipeline and standardized preprocessing.

2. The choice of a Switchable CycleGAN is appropriate for unpaired medical images. However, the manuscript should explain why this specific architecture is better for preserving clinical features than standard models. Generative models can sometimes create artificial patterns or hallucinations. Please discuss how you ensured that the enhancement process did not alter the actual pathology of the liver.

We thank the reviewer for this important comment. The Switchable CycleGAN uses a single shared generator with AdaIN-based modulation, which constrains bidirectional translation within a unified feature space and reduces the risk of structural drift compared with the conventional two-generator CycleGAN. Cycle-consistency and identity losses further discourage unnecessary structural changes, while AdaIN mainly adjusts feature statistics rather than generating new spatial structures. To ensure pathology was not altered, we conducted quantitative structure-preservation analyses (patch-wise SSIM and GLCM metrics) These points have been clarified in the Discussion section.

3. The manuscript presents only successful enhancement cases. To provide a balanced clinical assessment, please include representative failure-case images showing when enhancement is suboptimal or counterproductive, as well as a quantitative analysis of failure patterns (e.g., severe artifacts, obesity, heterogeneous parenchyma). Please also provide clinical guidelines on when not to apply the algorithm. This is critical for safe clinical deployment.

Thank you for comments. Representative failure-case images have been added. However, there are limitations to performing a detailed quantitative analysis of failure patterns, as the two reviewers evaluated image quality using a 5-point scale, which is inherently semi-qualitative. For this algorithm, we believe that prioritizing adaptation and modification according to each ultrasound system is necessary before more robust quantitative failure analysis can be conducted.

4. The validation set includes 100 cases but only 22 patients are in the non-CLD group. This small sample size for the control group may limit the statistical power of your findings. It is important to prove that the algorithm does not create false cirrhosis-like textures in healthy livers. I suggest adding more control cases or addressing this limitation more clearly. Additionally, for the LC diagnosis comparison (n = 47), please provide post-hoc power analysis for the observed sensitivity improvement (53.2 -> 66.0%, reviewer 1).

Thank you for your comments. We have described the limitation of the small sample size of the non-CLD group in the Discussion section. In addition, post-hoc power analysis showed that the post-hoc power for the sensitivity improvement observed by Reviewer 1 (53.2 → 66.0%) was approximately 0.28.

5. This study uses only one device pair (12-year-old Aloka -> 4-year-old Canon). The model essentially performs device-to-device translation rather than generalizable quality enhancement. Please discuss expected performance on images from other manufacturers and whether the approach requires retraining for each device pair. Please also address the feasibility of multi-vendor training or transfer learning. This limitation substantially affects the clinical applicability of your method.

We appreciate the reviewer’s comment regarding generalizability. We agree that the current model was trained on a single device pair and primarily performs device-to-device translation. Because ultrasound image characteristics vary across vendors, direct deployment to unseen systems may require retraining or fine-tuning. However, as the proposed framework is fully unsupervised and does not require paired data, adaptation to new vendors can be achieved using relatively small unpaired datasets. Multi-vendor training is also feasible through a multi-domain translation framework or transfer learning. We have clarified these limitations and potential solutions in the revised Discussion section.

6. The comparison with CT or MRI was performed within a six-month window. This time gap is relatively long because liver conditions can progress or change during that period. Please explain the rationale for this timeframe. Also, the manuscript lacks a description of the CT and MRI protocols used as the reference standard.

We thank the reviewer for this important comment. The six-month interval was selected to reflect routine clinical surveillance practice in chronic liver disease, where CT or MRI is typically performed every 3–6 months. Given the retrospective design, strict temporal matching was limited by available data. The evaluated conditions (liver cirrhosis, fatty liver, and focal lesions) are generally chronic structural abnormalities that do not rapidly change, although minor progression cannot be excluded. We have clarified this limitation in the revised manuscript. We have also added detailed descriptions of the CT and MRI acquisition protocols, including contrast phases and diagnostic criteria, in the Methods section.

7. Please add page and line numbers.

Thank you for the comment. We have added it.

8. Consider merging redundant sections (e.g., Introduction vs. Background) and tighten the narrative.

Thank you for this suggestion. Rather than removing the Background section, we revised the manuscript by reducing redundancy between the Introduction and Background while preserving key references. The Introduction now focuses on clinical motivation, knowledge gaps, and study objectives, whereas the Background has been condensed to provide a concise review of prior work relevant to ultrasound image enhancement and image-to-image translation.

9. Standardize abbreviations and define each once (avoid re-defining common terms).

Thank you for this comment. We have revised the manuscript to standardize the use of abbreviations throughout the text.

10. Fix table numbering and cross-references throughout.

Thank you for this comment. We have carefully revised the manuscript to standardize table numbering and cross-references throughout the text.

11. Check reference numbering using a reference manager.

We thank the reviewer for this comment. We have carefully reviewed all references using a reference management tool and corrected any numbering inconsistencies.

12. Clean up formatting issues (extra spaces, inconsistent capitalization, inconsistent singular/plural such as “test dataset(s)”).

Thank you for this suggestion. We carefully revised the manuscript to correct formatting inconsistencies, including extra spaces, capitalization, and singular/plural usage.

13. Make figure legends self-contained and ensure they match figure numbering.

We thank the reviewer for this suggestion. We have revised all figure legends to make them fully self-contained. We have also carefully checked and corrected figure numbering to ensure consistency between the text and figure captions.

14. Please report the inference time per image and discuss the computational requirements for potential real-time deployment during actual scanning procedures.

Thank you for this important comment. We have added the inference time per image and computational requirements to the Methods section and discussed the feasibility of real-time deployment in the Discussion. The proposed CycleGAN-based model processes a single ultrasound image in approximately 30–40 [ms] on an NVIDIA RTX 3090 GPU, corresponding to 25–30 FPS under single-image inference conditions, suggesting potential applicability for real-time enhancement during clinical ultrasound scanning.

Thank you again for the valuable comments of the reviewers. We did our best for the revision to enhance our manuscript's quality. We hope that the revised version of the manuscript meets with your and reviewer’s approval and will be considered for publication in the ***PLOS ONE***

Sincerely yours,

Joo Hyeok Choi, MD; Jaeyoung Huh PhD; Eun Sun Lee, MD, PhD
